# Assessing Complexity in Physiological Systems through Biomedical Signals Analysis

**DOI:** 10.3390/e22091005

**Published:** 2020-09-09

**Authors:** Paolo Castiglioni, Luca Faes, Gaetano Valenza

**Affiliations:** 1 IRCCS Fondazione Don Carlo Gnocchi, 20148 Milan, Italy; 2 Department of Engineering, University of Palermo, 90128 Palermo, Italy; luca.faes@unipa.it; 3 Department of Information Engineering and Research Center “E. Piaggio”, University of Pisa, 56122 Pisa, Italy; g.valenza@ing.unipi.it

**Keywords:** entropy, multifractality, multiscale, cardiovascular system, brain, information flow

The idea that most physiological systems are complex has become increasingly popular in recent decades. Complexity is now considered a ubiquitous phenomenon in physiology and medicine that allows living systems to adapt to external perturbations preserving homeostasis. Complexity originates from specific features of the system, like fractal structures, self-organization, nonlinearity, the presence of many interdependent components interacting at different hierarchical levels and at different time scales, as well as interconnections with other systems through physiological networks. Biomedical signals generated by such physiological systems may carry information on the system’s complexity, which may be exploited to detect physiological states, to monitor the health conditions over time, or to predict pathological events. For this reason, the more recent trends in the analysis of biomedical signals are aimed at designing tools for extracting information on the system complexity from the derived time series, like continuous electroencephalogram and electromyogram recordings, beat-by-beat values of cardiovascular variables, or breath-by-breath measures of respiratory variables.

This Special Issue collects 16 scientific contributions on the rapidly evolving field of time series analysis for evaluating the complex dynamics of physiological systems. To provide the general reader with a broad vision of this wide and articulated topic, this Special Issue not only called for novel methodological approaches devised to improve the existing complexity quantifiers, or novel applications of complexity analyses in physiological or clinical scenarios, but also for review papers describing the state of the art of the complexity methods in specific areas of clinical and biomedical research. 

In this regard, the Special Issue includes two reviews addressing particularly relevant clinical topics. The paper by Sun et al. [1] revises the studies on Alzheimer’s disease that quantified complexity alterations in the brain signals (electro- and magneto-encephalography or functional magnetic resonance imaging). The review points out a loss of signal complexity in the Alzheimer patients that might represent a biomarker of their functional lesions, useful in the diagnosis of the disease and in the quantification of brain dysfunction. The paper of Rampichini et al. [2] reviews the studies on the complexity analysis of the surface electromyography to detect the onset of fatigue in exercising muscles, an issue of great interest in physiology, pathophysiology, training, and rehabilitation. For each complexity index, the authors summarized its meaning, the estimation algorithms, and the results of the studies that applied it.

The novel methodological approaches that the readers will find in this Special Issue regard the theoretical aspects of the evaluation of entropy and information flow. The desired characteristic for any entropy estimator is relative consistency, in most applications assumed to make meaningful comparisons by setting specific values of the estimator parameters (like a given embedding dimension and a given tolerance threshold). However, there are no formal proofs of this property for the popular sample entropy estimator. Zurek et al. [3] demonstrated that the relative consistency of sample entropy does not hold for a certain class of random processes and therefore suggest that biomedical studies should identify the regions of relative consistency before drawing conclusions based on a single set of parameters. Interestingly, they also indicated how to evaluate the relative consistency in real physiological signals, such as long-term heart rate series, with a computationally efficient algorithm. The consistency of sample entropy for heart rate time series also underlies the work of Zhao et al. [4]. The authors reported that the presence of irregularities in the cardiac contraction (premature or ectopic beats) importantly influenced the sample entropy estimator, even causing a loss of its relative consistency, and address this problem proposing a new way to set the tolerance threshold. Furthermore, Velasquez-Martinez et al. [5] presented a new entropy estimator based on vector-quantized patterns, less sensitive to noise than sample and fuzzy entropy, to detect the event-related de-synchronization and synchronization of brain signals for applications in the field of brain–computer interfaces. 

Entropy measures reflect the level of information carried by the signals and its changes in time, and the assessment of information dynamics is the topic of the contribution of Antonacci et al. [6]. Following the paradigm of network physiology, a complex system is studied dissecting the information generated, stored, and modified in, or transferred to, target subsystems. Entropy estimation based on linear parametric modeling requires a high ratio between the number of data points available and the number of model parameters, a condition rarely occurring in biomedical applications. To overcome this limit, the authors propose a new estimation approach demonstrating its potential on real cardiovascular, respiratory, and brain signals simultaneously recorded during mental tasks.

Most of the contributions to this Special Issue (10 papers) regard novel applications of complexity-based analyses in physiological or clinical settings. Overall, this Section presents a wide spectrum of complex methods that investigate the entropic properties, the multifractal structures, or the presence of self-organized criticality in the studied physiological systems. This Section touches upon three areas of physiological applications: the cardiovascular system, the central nervous system, and the heart–brain interactions.

Regarding the cardiovascular system, the work of Makowiec and Wdowczyk [7] explores patterns of heart rate variability from night-time electrocardiographic recordings, making use of entropic measures and machine learning methods. Their exploratory analysis indicates that five main factors, possibly associated with vagal and cardiac sympathetic outflows, autonomic balance, homeostatic stability, and humoral effects, drive the complex heart rate dynamics. Heart-rate entropy analyses are also considered in the paper of Monteiro-Santos et al. [8], who derived fetal heart rate series from cardiotocographic signals recorded on the mothers’ abdomen between 30 and 35 gestational weeks. Their results indicate that the complexity measures of fetal heart rate contribute to the prediction of labor, a finding that opens the possibility to improve the assessment and care of the fetus and the mother. Xiao et al. [9] considered a second cardiovascular signal in addition to the electrocardiogram: the finger photoplethysmogram. They derived the beat-by-beat series of heart rate and pulse wave amplitude and quantified the similarity of the two series by the percussion entropy. Their results suggest that this entropy measure may distinguish diabetic patients with a satisfactory control of blood glucose from those with poor control, highlighting the feasibility of assessing autonomic dysfunctions of clinical relevance by the percussion entropy. Also Faini et al. [10] considered a second cardiovascular signal in addition to the electrocardiogram: the finger arterial pressure. These authors calculated the multiscale sample entropy of the heart rate series and of the series of systolic and diastolic arterial pressure in volunteers at sea level and at high altitude and explained the alterations observed at high altitude by the increased chemoreflex sensitivity induced by hypoxia. Since high altitude is a model of some pathological states that occur at sea level, like heart failure, their work provides a possible interpretation for the alterations in the multiscale entropy of cardiovascular signals that may be observed in cardiac patients.

Entropy is not the only complexity feature of the cardiovascular system addressed in this Special Issue. The work of J.O. Fortrat [11] investigates the presence of self-organized criticality evaluating whether bradycardic heart-rate sequences follow a Zipf’s law during the head-up tilt test. Results support the hypothesis of cardiovascular self-organized criticality and provide evidence of a different distribution of bradycardic sequences in the participants who experienced syncope symptoms during the test. Furthermore, cardiovascular multifractality is the topic of the paper by Castiglioni et al. [12] that quantifies the multifractal–multiscale structure of the heart-rate and blood-pressure series, revealing night/day modulations of nonlinear fractal components at specific temporal scales. The work suggests that the multifractal–multiscale approach improves the clinical value of the 24 h analysis of blood pressure and heart rate variability.

Two studies apply complexity analyses on brain signals. The paper by Jia and Gu [13], based on functional magnetic resonance imaging, aims at describing the structure of functional networks in the brain from measures of the dynamic functional connectivity (assessed as the time series of correlation values between the blood-oxygenation level-dependent signals of distinct brain regions calculated over a sliding window). The authors classified the sample entropy measured for each dynamic functional connectivity series using a machine learning method, and found six clusters that represent as many functional networks of the human brain, contributing to a better understanding of the complexity of the brain networks. The paper by Ghouse et al. [14] focuses on functional near-infrared spectroscopy measurements aiming to calculate sample, fuzzy, and distribution entropy of the time series of hemoglobin concentration during different mental tasks. The results suggest that complexity-based approaches uncover meaningful activation areas that complement those identified by traditional analyses.

Finally, two contributions to this Special Issue investigate "brain–heart interactions". The paper by Deschodt-Arsac et al. [15] demonstrates that five weeks of a biofeedback training able to reduce stress and anxiety increases the multiscale entropy of heart rate during a stressful cognitive task. The results support the hypothesis that the adopted biofeedback training restores a healthy response to stress consisting of an increased heart rate complexity through mechanisms of neurovisceral integration. Blons et al. [16] measure multiscale entropy from signals representative of different neurophysiological networks: the heart rate and the postural sway of the center of pressure. The study demonstrates an increase in the multiscale entropy of both signals during cognitive tasks, highlighting that in healthy individuals an increased complexity of the neural structures involved in the functional brain–heart interplay may facilitate the adaptability of the central and peripheral control to face demanding tasks. 

We hope that the papers collected in this Special Issue will inspire future methodological and clinical works advancing this fascinating area of research.
